# Genital Cytomegalovirus Replication Predicts Syphilis Acquisition among HIV-1 Infected Men Who Have Sex with Men

**DOI:** 10.1371/journal.pone.0130410

**Published:** 2015-06-10

**Authors:** Sara Gianella, Davey M. Smith, Eric S. Daar, Michael P. Dube, Andrea Lisco, Christophe Vanpouille, Leonid Margolis, Richard H. Haubrich, Sheldon R. Morris

**Affiliations:** 1 University of California, San Diego, La Jolla, California, United States of America; 2 Veterans Affairs San Diego Healthcare System, San Diego, California, United States of America; 3 Los Angeles Biomedical Research Institute at Harbor-UCLA Medical Center, Torrance, California, United States of America; 4 University of Southern California Keck School of Medicine, Los Angeles, California, United States of America; 5 National Institute of Allergy and Infectious Diseases, National Institutes of Health, Bethesda, Maryland, United States of America; 6 National Institute of Child Health and Human Development, National Institutes of Health, Bethesda, Maryland, United States of America; Fudan University, CHINA

## Abstract

**Objective:**

Sexually transmitted infections (STI) are common among HIV-infected men who have sex with men (MSM). While behavioral factors are important in STI acquisition, other biological factors such as immune modulation due to chronic viral infection may further predispose to STI acquisition.

**Design:**

Post Hoc analysis including data collected over 12 months of follow-up from 131 HIV-infected MSM receiving antiretroviral therapy and screened for incident bacterial STI every 3 months.

**Methods:**

Genital secretions collected at baseline were used to measure herpesvirus replication and inflammatory cytokines. Baseline predictors of STI were determined using survival analysis of time to incident STI.

**Results:**

All participants were seropositive for cytomegalovirus (CMV), and 52% had detectable genital CMV at baseline. Thirty-five individuals acquired STI during follow-up, sometimes with multiple pathogen (17 syphilis, 21 gonorrhea, 14 chlamydia). Syphilis acquisition was associated with genital CMV replication at baseline (19.1% CMV-shedders versus 4.8% non-shedders, *p*=0.03) and younger age (*p*=0.02). Lower seminal MCP-1 was associated with higher seminal CMV levels and with syphilis acquisition (*p*<0.01). For syphilis acquisition, in multivariable Cox-Proportional Hazard model adjusted hazard rates were 3.56 (95%CI:1.00–12.73) for baseline CMV replication and 2.50 (0.92–6.77) for younger age.

**Conclusions:**

This post hoc analysis suggest that CMV-associated decrease in seminal MCP-1 levels might predispose HIV-infected MSM to syphilis acquisition, but not other STI. Future studies should determine underlying mechanisms and if a causal association exists.

## Introduction

Rates of sexually transmitted infections (STI) are considerably higher among men who have sex with men (MSM) than in other risk groups in the United States, and this is particularly true for syphilis (*Treponema pallidum*) [1]. Syphilis is also one of the most consequential STI because it is associated with long-term complications, such as neurosyphilis [2]. From 2005 to 2013, the number of primary and secondary syphilis cases in the United States rose from 2.9 to 5.3 per 100,000 population [3]. Remarkably, MSM accounted for over 80% of all new syphilis cases in areas reporting in 2012 [3], and among those MSM with syphilis, about half were also infected with HIV [4]. It is remarkable that despite HIV prevalence in the general population being <1%, HIV infected individuals account for almost 50% of all primary and secondary syphilis, suggesting that HIV-infected individuals are indeed paticularly susceptible to aquisition of syphilis.

The reasons for this high burden of syphilis among HIV-infected MSM are not entirely clear. Behavioral risk and sexual networks play an important role, including use of recreational drugs and higher number of sexual partners [1, 5], but other biological factors may also be at play, including immunomodulatory mechanisms secondary to the presence of other infections. Multiple pathogens often co-infect the same host and can influence each other’s dynamics and replication [6–9]. For example, genital co-infections such as gonorrhea, chlamydia, or human herpesviruses (HHV) are associated with increased HIV RNA genital shedding and HIV transmission [10–13], and syphilis, HHV, gonorrhea, trichomonas, and other infections are associated with HIV acquisition [14–21]. Presence of HSV-2 has been previously associated with bacterial vaginosis in HIV-infected women [22]. Several recent studies have suggested that HHV in the mouth cavity can play a role in the development of periodontitis and peri-implantitis as oral colonization with cytomegalovirus (CMV) and Epstein Barr virus (EBV) has been associated with pathogenic bacterial colonization, including *spirochetes* [15, 23, 24]. In this study, we performed a post-hoc analysis to investigate the relationship between HHV infection and acquisition of bacterial STI in a cohort of HIV-infected MSM on antiretroviral therapy (ART). In our primary analysis we investigated if the presence of asymptomatic seminal CMV DNA replication at baseline was associated with acquisition of syphilis, gonorrhea or chlamydia during the subsequent 12 months of follow-up.

## Materials and Methods

### Participants, samples, and clinical laboratory tests

The studies were conducted with appropriate written consent and were approved by the Human Research Protections Program at University of California San Diego, Los Angeles Biomedical Research Institute at Harbor-UCLA Medical Center, and the University of Southern California.

A total of 179 participants were prospectively enrolled and followed in the parent California Collaborative Treatment Group (CCTG) 592 study, which was an internet-based behavioral intervention study of HIV-infected MSM at high risk for STI. At baseline, there were 131 participants receiving ART with HIV RNA <500 copies/ml in blood plasma, and thus eligible for this post-hoc analysis. At baseline and every 3 months participants received extensive STI testing consisting of throat, rectal, and urine samples for *Neisseria gonorrhoeae* and *Chlamydia trachomatis* using transcription-mediated amplification (TMA) (Genprobe Aptima, San Diego), and completed a computer-assisted self-reported interview for sexual risk behavior, drug use, and adherence to ART in the previous month. Additionally, we evaluated active syphilis infection using rapid plasma reagin (RPR) titers with *Treponema pallidum* particle agglutination assay (TPPA) confirmatory testing and clinical history. All STI incidents were adjudicated by an independent endpoint review committee of 3 physicians with expertises in infectious disease to determine if a case was considered as a new infection. Each timepoint was defined as 1) no syphilis, 2) serofast status of previously treated syphilis, 3) incident syphilis.

Baseline RPR was interpreted in relation to previously measured RPR titers (when available), treatment history, and relevant clinical information to determine if a new syphilis case was present. In case of a positive RPR at baseline, incident syphilis during follow-up was defined by a 4-fold increase in RPR titer (according to standard Sexually Transmitted Diseases Treatment Guidelines [25]). If baseline RPR was negative, then any new positive RPR titer was considered as a new syphilis infection.

As part of the study protocol, blood and semen samples were collected at baseline for all participants [12].

Semen was collected and processed as previously described [26, 27]. We measured blood CD4+ T lymphocyte subsets using flow cytometry (CLIA certified laboratories) and HIV RNA levels in blood plasma using the Amplicor HIV Monitor Test (Roche Molecular Systems Inc.).

### Herpesvirus DNA and HIV RNA extraction and quantification from seminal plasma

We used real-time PCR to measure levels of HIV RNA and different HHV in semen (CMV, EBV, herpes simplex viruses (HSV) types 1 and 2, and HHV types 6, 7, and 8) [27, 28].

### Multiplex-bead-array assay for cytokines/chemokines quantification

Selected markers of genital inflammation (monocyte chemotactic protein [MCP]-1, interleukin [IL]-6, tumor necrosis factor [TNF]-α, Interferon-γ, regulated on activation normal T cell expressed and secreted [RANTES], and Interferon-γ induced protein [IP]-10) were measured in seminal plasma at baseline for a subset of 110 subjects when enough seminal plasma was available for additional testing [7].

### Statistics

Statistical analyses were performed with SAS (version 9.2). For this post-hoc anaysis, viral load variables were transformed to logarithm-base ten values. We tested continuous variables for normality with the Shapiro-Wilk test, and if they failed to be normal we compared them using nonparametric tests (for CMV serology, cytokine levels) or dichotomized them using quartiles (age and partner number). We performed comparisons between groups (e.g., seminal CMV shedding versus non-shedding, age younger versus older than 40 years) using the Fisher-exact test (for sparse categorical variables), t-test (for continuous, normally distributed variables), or the Mann Whitney U test (for continuously, non-normally distributed variables). Correlation between continuous variables was tested with the Spearman rank correlation. For the outcome of incident syphilis, we used Cox Proportional Hazard models in univariable and multivariable regression to adjust for possible confounders.

In addition to CMV DNA seminal shedding (primary objective), additional variables evaluated included behavioral factors (number of sex partners, number of anal sex acts, use of methamphetamine and other drugs), blood and seminal plasma HIV RNA levels, current and nadir CD4 and CD8 T cell count, genital shedding of other HHV (EBV, HSV, HHV-6/-7/-8), and soluble markers of genital tract inflammation (MCP-1, IL-6, TNF-α, Interferon-γ, RANTES, and IP-10 in baseline seminal plasma). The primary hypothesis tested was whether the presence of genital CMV shedding at baseline was associated with acquisition of syphilis, gonorrea or chalmydia over the subsequent 12 months.

## Results

### Study participants’ demographics and clinical data

Study participants (*n* = 131) were HIV-infected MSM receiving ART and having HIV RNA<500 copies/ml in blood plasma at enrollment (the median time between baseline and blood HIV RNA level was 32 days, with interquartile range [IQR] of 13–68 days). The majority (84.0%) had <50 HIV RNA copies/ml in blood plasma. The median CD4 T cell count was 604 cells/μL (IQR: 414–761). Participants were predominantly Caucasian (*n* = 84, 64%), and the median age was 47 years (IQR: 39–52). Regarding specific ART information, 76.7% were on a regimen including tenofovir, 39.3% were on a regimen including a non-nucleoside reverse transcriptase inhibitor (NNRTI), 55.4% were on a regimen including a protease inhibitor (PI), and 18.8% were on a regimen including an integrase inhibitor. Self-reported levels of ART adherence during the last month were >90% for 87% of the subjects. Nighty-seven participants (74.05%) completed the study (month 12 follow-up and 112 participants (85.5%) completed at least month 9. Characteristics and demographics of the study participants are summarized in Table 1.

**Table 1:** Demographics and co-infections at baseline.

Characteristics at Baseline		n (%)
Participants		131 (100)
MSM		131 (100)
Age (years); median (IQR)		47 (39–52)
Race, n (%):	Caucasian	84 (64.1)
	Black	41 (31.3)
	Other	6 (4.6)
Hispanic Ethnicity (n,%)		43 (32.8)
HIV RNA <50 copies/ml; n (%)		110 (84.0)
HIV RNA 50–500 copies/ml; n (%)		21 (16)
≥90% adherence past month n (%)		115 (87.8)
CD4+ cell counts/ul; median (IQR)		604 (414–761)
Detectable HIV RNA in semen; n (%)		15 (11.5)
HIV in semen log10 copies/ml; median (range)		125 (50–195)
Unprotected anal sex acts past month; median (range)		0 (0–2)
Methamphetamine use; n (%)		17 (13.3)
Any drug use; n (%)		46 (35.9)
Number Male Sexual Partners past month; median (range)		3 (1–6)
**Bacterial Sexually Transmitted Infections at Baseline**		
Urethra; n (%)	Gonorrhea	0 (0)
	Chlamydia	1 (0.8)
Rectum; n (%)	Gonorrhea	4 (3.1)
	Chlamydia	5 (3.8)
Throat; n (%)	Gonorrhea	5 (3.8)
	Chlamydia	1 (0.8)
Syphilis (new cases); n (%)		1 (0.8)
Any psitive RPR at baseline; n (%)		27 (20.8)
**Herpesviruses Shedding**		
Any detectable HHV DNA; n (%)		84 (64.1)
Any detectable HSV (1 or 2) DNA; n (%)		3 (2.3)
Any detectable CMV DNA; n (%)		68 (51.9)
Any detectable EBV DNA; n (%)		36 (27.5)
Any detectable HHV-6 DNA; n (%)		9 (6.9)
Any detectable HHV-7 DNA; n (%)		11 (8.4)
Any detectable HHV-8 DNA; n (%)		4 (3.1)

Legend: n (%): number (percentage) of participants; MSM: men who have sex with men; HIV: Human immunodeficiency virus; ART: antiretroviral therapy; IQR: interquartile range; HHV: Human Herpesviruses; CMV: cytomegalovirus; EBV: Epstein-Barr virus; HSV-1 and -2: Herpes simplex virus type 1 and type 2; HHV-6/-7/-8: human herpesvirus type 6/type 7/type 8.

### Genital tract infections at baseline

At baseline, 36 (27.5%) individuals had a bacterial STI that included i) chlamydia in the rectum (5, 3.8%), urethra (1, 0.8%) and throat (1, 0.8%); ii) gonorrhea in the rectum (4, 3.1%) and throat (5, 3.8%); and (iii) new syphilis (1, 0.8%) and any RPR positivity, i.e. RPR ≥1:1, (27, 20.6%). As determined from clinical history 26 of the positive RPR found at baseline were determined to be either known infection post-treatment or serofast status. Additionally, 84 (64.1%) had DNA present from at least one HHV in their seminal plasma, including HSV-1/-2 (3, 2.3%), CMV (68, 51.9%), EBV (36, 27.5%), HHV-6 (9, 6.9%), HHV-7 (11, 8.4%), and HHV-8 (4, 3.1%) [12] (Table 1). There were no statistical differences in the rates of HHV shedding, combined or individually, between patients treated with tenofovir, NNRTI, PI, or integrase inhibitors.

During the 12 months of follow-up, 35 subjects (26.7%) acquired new bacterial STI, of whom 11 participants had 2 STI events and 1 participant had 3 STI events, for a total of 48 newly diagnosed STI, as decided by the independent outcome committee. Of these STI, 17 were incident syphilis cases from 16 individuals during study follow-up (one person had syphilis twice defined by a second time point with a four fold increase in RPR, but this person was counted only once in the subsequent analysis), and 23 subjects (17.6%) acquired a bacterial STI that was not syphilis, i.e., gonorrhea in the rectum (*n* = 8), urethra (*n* = 2), or throat (*n* = 11), and chlamydia in the rectum (*n* = 12) or urethra (*n* = 2).

### Predictors of acquisition of syphilis during follow-up

**Fig 1:**
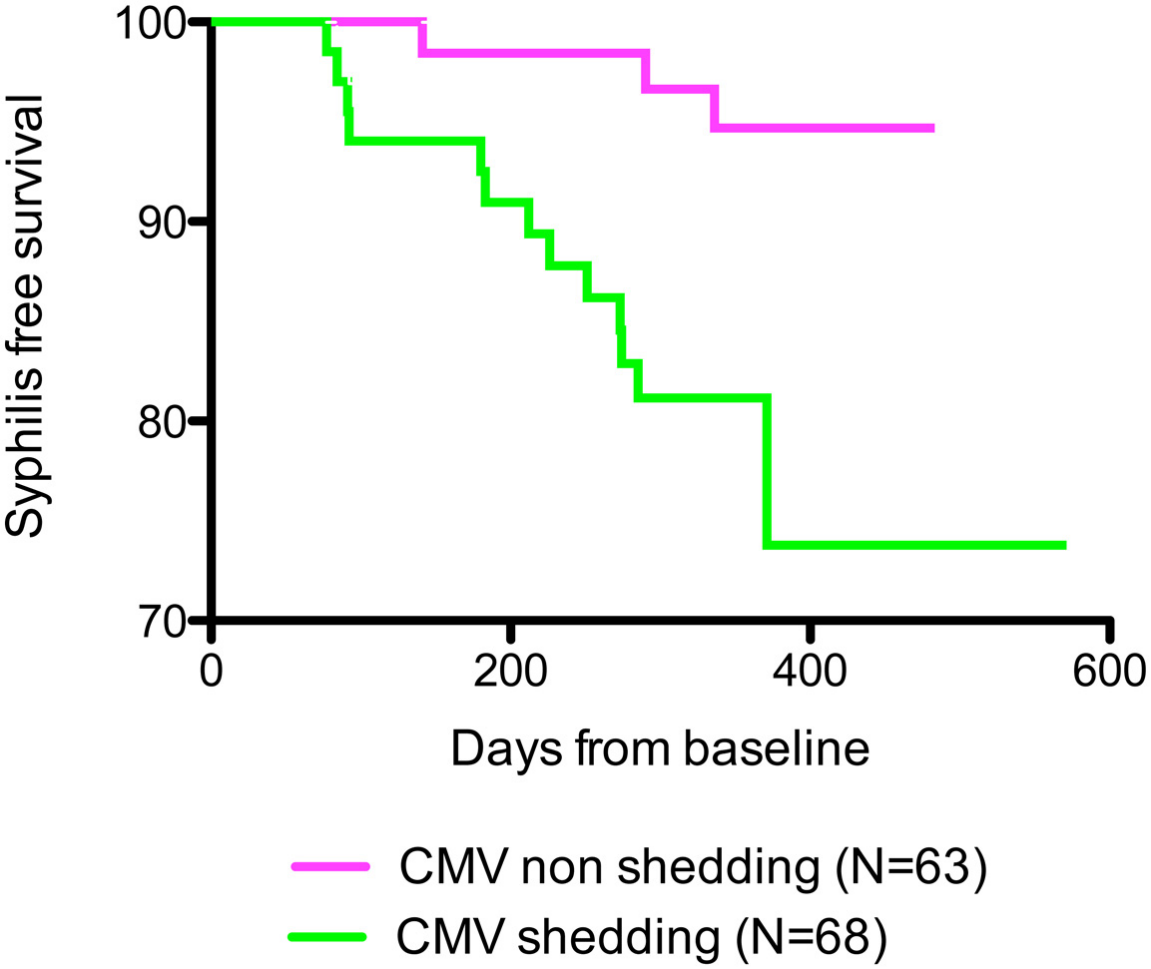
Kaplan Meier of Syphilis Cases. Kaplan Meier curve of time to incident syphilis during follow-up in subjects with detectable seminal CMV DNA (in green, N = 68) and without detectable seminal CMV DNA (in pink, N = 63) at baseline.

In our cohort, the main baseline predictors of acquiring syphilis over the course of the study according to Cox-Proportional Hazard regression were baseline genital shedding of CMV and younger age (<40 years old). Specifically, of the 68 individuals shedding CMV in semen at baseline, 13 (19.1%) acquired syphilis over the next 12 months, compared with only 3 of the 63 participants who were not shedding CMV at baseline (4.8%) (*p* = 0.03, Fig 1). Additionally, 8 of the 36 MSM who were younger than 40 years (22.2%) acquired syphilis, compared with 8 of 95 (8.4%) of the MSM who were older than 40 years (*p* = 0.02). Since younger age was also marginally associated with CMV shedding (66.7% of young MSM were shedders compared with 46.3% of older MSM, *p* = 0.05), we performed a multivariate analysis that included CMV shedding and younger age. In this multivariate model, CMV shedding had an adjusted hazard rate for syphilis acquisition of 3.56 (95% CI: 1.00–12.73) and younger age had a hazard rate of 2.50 (95% CI: 0.92–6.77). Table 2 summarizes the analysis for all included variables. HIV seminal shedding and the presence of syphilis at baseline were not associated with syphilis acquisition during follow-up.

**Table 2:** Factors associated with incident syphilis.

Factor	Syphilis; n (%)	HR	P-value	Adjusted HR
Any detectable seminal CMV DNA	13 (19.1)	4.14	**0.03**	3.56 (1.00–12.73)
No detectable seminal CMV DNA	3 (4.8)			
Younger age (<40 years)	8 (22.2)	3.11	**0.02**	2.50 (0.92–6.77)
40 or older	8 (8.4)			
Caucasian/Non-Hispanic, n (%)	8 (18.6)	2.14	0.13	
CD4+ T-cells/μl, mean (95% CI)	591.5 (324–735)	1	0.35	
Blood log10 HIV RNA <50 copies/ml	13 (11.8)	0.86	0.82	
Any detectable HIV RNA in semen	3 (20.0)	1.92	0.31	
Any detectable HSV-1/HSV-2 DNA	0 (0)	0	0.99	
Any detectable EBV DNA	6 (16.7)	2.27	0.44	
Any detectable HHV-6 DNA	2 (12.5)	3.4	0.28	
Any detectable HHV-7 DNA	3 (27.3)	3.92	0.06	
Any detectable HHV-8 DNA	2 (22.2)	2.05	0.19	
Baseline syphilis	5 (18.5)	1.07	0.26	
Number of male partners past month (>6)	5 (20.8)	2.1	0.18	
Any unprotected anal sex acts past month	5 (9.8)	0.67	0.46	
Any methamphetamine use	3 (17.7)	1.88	0.33	
Any illicit drug use other than marijuana	7 (15.2)	1.54	0.39	

Legend: n (%): number (percentage) of participants; HR: hazard ratio; IQR: interquartile range; 95% CI: 95% confidence intervals; HIV: Human immunodeficiency virus, ART: antiretroviral therapy; CMV: cytomegalovirus; EBV: Epstein-Barr virus; HSV-1 and -2: Herpes simplex virus type 1 and type 2; HHV-6/-7/-8: human herpesvirus type 6/type 7/type 8. In **bold:** significant *p*-values (*p*<0.1).

The presence of detectable seminal DNA for HHV-6, HHV-7, and HHV-8 at baseline was also associated with higher rates of incident syphilis, but these associations did not reach statistical significance, likely because of the lower prevalence. In combined shedding results of all tested genital HHV (including HSV-1, HSV-2, CMV, EBV, HHV-6, HHV-7, and HHV-8), 15 (93.8%) of all participants who acquired syphilis during follow-up had at least one HHV detectable in semen at baseline. The age-adjusted hazard rate for having at least one HHV shedding in semen to predict syphilis acquisition was 7.59 (95% CI: 0.99–58.09).

### Predictors of acquisition of gonorrhea and chlamydia during study follow-up

The only baseline predictor of acquisition of STI other than syphilis (i.e. chlamydia or gonorrhea at any anatomic site) was higher number of sexual partners (>6 in the past month). In fact, 9 of 24 (37.5%) MSM within the highest quartile of numbers of partners had either gonorrhea or chlamydia, compared with 14 of 106 MSM (13.2%) with lower numbers of partners (*p* = 0.02). Of all other factors, genital CMV shedding showed a weak positive trend towards significance, since 16 out of the 68 CMV shedders (23.5%) acquired either gonorrhea or chlamydia compared with 7 out of 63 of CMV non-shedders (11.1%) (*p* = 0.10).

### Association of bacterial and viral co-infections with genital Inflammation

**Fig 2:**
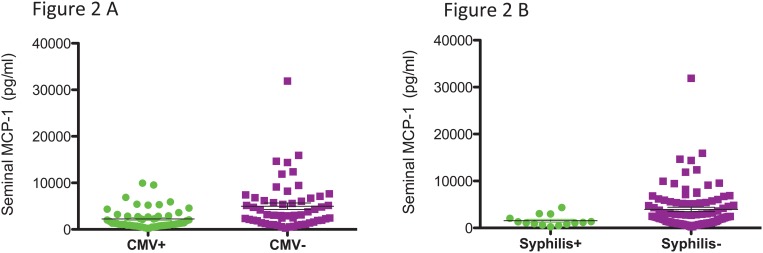
Genital MCP-1 levels, CMV shedding and syphilis acquisition. CMV shedding (panel A) and syphilis acquisition (panel B) were both associated with LOWER seminal MCP-1 levels (Mann Whitney test).

To investigate possible inflammatory mechanisms connecting CMV shedding and acquisition of syphilis, we measured levels of selected cytokines and chemokines (i.e., MCP-1, IL-6, TNF-α, Interferon-γ, RANTES, and IP-10) in seminal plasma. We found that participants with detactable CMV in the genital tract at baseline had significanlty lower levels of MCP-1 in semen compared to non CMV shedders (median of 1351 [IQR: 839–2847] vs. 3108 [1570–6722] pg/ml, p = 0.0006, Fig 2A). Also, participants who acquired syphilis had significantly lower levels of seminal MCP-1 at baseline than those who did not acquire syphilis (median 1,141.6 pg/ml [IQR: 819.02046.4] compared with 2,436.4pgI/ml [1084.5–5339.4], *p* = 0.01, Fig 2B). The level of this cytokine was lower in participants who acquired gonorrhea or chlamydia, but did not reach statistical significance (*p* = 0.06). None of the other tested cytokines was associated with CMV shedding or syphilis acquisition.

## Discussion

Syphilis acquisition is very common among HIV-infected MSM [3]. While this observation has been generally attributed to high-risk sexual behavior and close sexual networks [1], other biological factors that may link these two infections have not been adequately investigated and could represent alternative targets for intervention. Here, we performed a post-hoc analysis to investigate alternative predictors of incident syphilis and other bacterial STI among HIV-infected MSM on effective ART. In this study, we found a significant association between presence of CMV shedding at baseline and new syphilis infections over the next 12 months, and the hazard rate was almost 4 times that of non-CMV-shedders.

The main confounder for this effect was age, with younger age associated with both CMV shedding and acquisition of syphilis; however, baseline CMV shedding was still significantly associated with syphilis acquisition after adjustment for age in a multivariate analysis. When considering shedding of all HHV together (HSV-1/-2, CMV, EBV, HHV-/6-7/-8), we found that having detectable HHV at baseline in semen samples was associated with a risk of syphilis acquisition nearly 8 times higher than in those without any detectable HHV. This association was however, driven by CMV shedding, since none of the other HHV was significantly associated with syphilis acquisition individually. Presence of HIV seminal shedding and positive RPR titers at baseline was not associated with syphilis acquisition during follow-up. While tenofovir/emtricitibine can reduce acquisition and shedding of HSV-2 [29, 30], in our analysis we didn’t find any differential effects of ART regimens on any HHV shedding, likely because only a small fraction of our participants were not on a tenofovir/emtricitibine-containing regimen.

A high number of sexual partners was the only significant factor associated with acquisition of chlamydia or gonorrhea at any anatomic site.

Possible synergistic interactions between HHV and bacterial infections (including *spirochetes*) have been described before in different settings [15, 22–24]. For example, in the setting of periodontitis, detectable CMV and EBV in saliva has been associated with pathogenic bacterial colonization including *Treponema denticola*, which is a close relative of *Treponema pallidum* [15, 23, 24]. In the setting of organ transplantation, presence of CMV in the mouth has also been associated with overgrowth of gram-negative bacteria in oropharyngeal flora [31]. Although the mechanism of these associations is still unclear, it seems that replication of HHV (CMV and EBV in particular) could contribute to adhesion, penetration, and/or immune evasion of syphilis and other bacterial pathogens.

To investigate possible inflammatory mechanisms connecting CMV shedding and acquisition of syphilis, we measured levels of selected cytokines/chemokines in seminal plasma. This analysis found that individuals with detectable genital CMV DNA and those who acquired syphilis had significantly lower levels of MCP-1 in semen compared to those without CMV shedding and who did not acquire syphilis during follow-up. This is interesting since MCP-1 is a potent chemotactic factor for monocytes produced by many cell types including macrophages, fibroblasts, endothelial and epithelial cells in response to tissue damage and oxidative stress. In seminal plasma, MCP-1 is thought to play a role in immune surveillance and modulation of immune response [32, 33]. We found that higher CMV seminal shedding was associated with lower seminal concentration of MCP-1. These findings are in agreement with previous studies that demonstrated that MCP-1 and its cognate chemokine receptor CCR2 are targeted by CMV-mediated immune modulation. In fact, CMV downregulates MCP-1 production by directly inhibiting its transcription [34, 35] and encoding a viral chemokine receptor (US28) that can modulate MCP-1 functions in CMV-infected cells [36]. Although it is unclear how such an effect could result in an advantage for CMV *in vivo*, it is possible that the interference with the MCP-1/CCR2 axis could result in CMV immune escape and consequent expansion of the pool of CMV-infected cells. Conceivably these changes in the immunological milieu of the male genital tract could also affect the acquisition and replication of other infectious pathogens. In particular, since the MCP-1/CCR2 axis has also been recently found to be involved in cell-mediated clearance of treponemal infection [37, 38], we speculate that CMV-mediated altered recruitment and function of monocytes/macrophages via the MCP-1/CCR2 axis may in turn affect the early immune response to *Treponema pallidum* and therefore increase the probability of acquisition of this pathogen. We recognize that, since *Treponema pallidum* is usually acquired as a transcutanous or trans-mucosal infection, local CMV shedding and altered cytokines milieu in seminal plasma may not be directly associated to increased syphilis acquisition but might reflect CMV replication and altered innate immune response in the surrounding genital area [39].

This study has several limitations. First, it is an exploratory analyses based on a post-hoc model and therefore the significance of the reported p values should be interpreted with caution. Second, despite the high rate of incident STI in our cohort, these results should be confirmed in a larger prospective cohort including both HIV-infected and uninfected individuals. The observed association between CMV DNA shedding and incident syphilis might be confounded by an unmeasured factor. In particular, as discussed above, there could be a common HIV-related immune defect that could make individuals susceptible to both CMV reactivation and syphilis acquisition. We were not able to adjust for sexual networks with high rates of circulating CMV and syphilis. Possible surrogates of this could be age and race/ethnicity, since people with similar age and race/ethnicity are more likely to belong to the same sexual network [40]. While race/ethnicity was not associated with acquisition of STI, we found an association between younger age and both CMV shedding and syphilis acquisition. Nevertheless, the presence of CMV shedding at baseline remained an independent predictor of syphilis acquisition, after adjustment for age. Lastly, highly sexually active MSM with recurrent genital tract infections or episodes of CMV superinfections, could conceivably present both CMV shedding and increased syphilis episodes. Nevertheless, in our study we didn’t find any association between presence of STIs at baseline (including previous syphilis by any positive RPR) and subsequent acquisition of any bacterial STI.

In summary, almost all HIV-infected MSM are co-infected with CMV, and prevalence of CMV seminal shedding in these individuals is significantly higher than in HIV-uninfected controls [41]. A disproportionately high prevalence of syphilis acquisition is described among the same population. Because of this epidemiologic co-occurrence, we hypothesized that CMV replication could play a role in acquisition of syphilis among HIV-infected MSM. We found that CMV shedding was indeed associated with syphilis acquisition, with a hazard rate almost 4 times that of non CMV-shedders. Investigating possible mechanisms, we found that lower levels of MCP-1 were associated with both CMV shedding and syphilis acquisition. Future studies are needed to further delineate such mechanistic underpinnings and confirm direct connections between CMV shedding and syphilis susceptibility.
